# Editorial: Immunotherapy for hematological malignancies in children

**DOI:** 10.3389/fped.2023.1315218

**Published:** 2023-10-25

**Authors:** Yusi Zhou, Yang Su, Yutong Xu, Ming Shi

**Affiliations:** ^1^ Cancer Institute, Xuzhou Medical University, Xuzhou, China; ^2^ Center of Clinical Oncology, The Affiliated Hospital of Xuzhou Medical University, Xuzhou, China; ^3^ Jiangsu Center for the Collaboration and Innovation of Cancer Biotherapy, Xuzhou Medical University, Xuzhou, China

**Keywords:** immunotherapy, hematological malignancies, leukemia, car-t, blinatumomab

**Editorial on the Research Topic** Immunotherapy for hematological malignancies in children

Acute lymphocytic leukemia (ALL) is the most common malignant tumor in children, accounting for nearly a quarter of all pediatric cancer cases according to the immune phenotype (1). In the past, the treatment of leukemia included three stages: remission, induction, consolidation and maintenance of acute lymphocytic leukemia in children, lasting about 2–2.5 years. Most traditional chemotherapy drugs were developed before 1970. The best dose and scheme for combined chemotherapy is to adjust the dose based on tolerance, MRD response evaluation, individualized pharmacodynamics and pharmacogenomics research, but the use of the biological characteristics of leukemia cells obtained by genomic analysis is limited. And further increasing the intensity of conventional chemotherapy will be positively correlated with significant adverse reactions (2). Therefore, it is necessary to find new ways to improve the induction remission rate of relapsed/refractory leukemia (3).

In the past decade, we have made great progress in our understanding of the genetic and biological basis of children's acute lymphocytic leukemia (ALL). Incorporating effective immunotherapy into leukemia treatment will reduce the intensity of conventional chemotherapy, thus reducing related toxicity and further improving the survival and life quality of patients with autologous disease. Immunotherapy, such as Blinatumomab and CAR-T cell therapy, have been approved for relapsed/refractory B-ALL, changing the treatment environment for children with relapsed/refractory leukemia. Blinatumomab and CAR-T cell therapy showed excellent remission and controllable toxicity in children with relapsed/refractory leukemia. At present, the 5-year event-free survival rate of children with acute lymphocytic leukemia (ALL) has increased to more than 85% (4–6). However, the 5-year total survival rate of children with relapsed /refractory leukemia does not exceed 50%, and the duration of remission is limited (2).

Blinatumomab is a double-specific T cell conjugate agent for anti-CD3 and anti-CD19 fragments, which can connect human CD3 ^+^ T cells with CD19 ^+^ leukemia cells, triggering cytotoxic immune response and playing an anti-tumor role (7). Both CAR-T cells and Blinatumomab use antibodies or antibody fragments to direct T cells to specific tumor-related antigens to achieve targeted anti-tumor effects. The effectiveness of immunotherapy is higher than that of traditional chemotherapy. Compared with Blinatumomab, CAR-T cell therapy targeting CD19 seems to be more effective, which may be due to the additional advantage of CAR-T cell therapy activating T cells. CAR-T cell therapy has obvious advantages in children with all patients with extramedullary recurrence. However, Blinatumomab is safer because CAR-T cell therapy is more prone to high-level CRS and neurotoxicity. Adverse events related to immunotherapy mainly include CRS caused by antigen-specific T cell activation, resulting in the subsequent release of inflammatory cytokines and ICANS (8, 9).

In this research topic, a retrospective observational study by Wu et al. shows that Blinatumomab is safe and effective for the consolidation treatment of B-cell acute lymphoblast leukemia (B-ALL). The study included a total of 23 child patients. The results showed that Blinatumomab was effective and safe in the short term in child B-ALL patients with MRD^+^ or chemotherapy-related toxicity. (Wu et al.). The research results of Xie et al. and others further show that short-term Blinatumomab can not only achieve bone marrow relief in R/R BCP-ALL children, but also have fewer side effects in the treatment process, providing hope for long-term survival. In developing countries, short-course Blinatumomab can achieve satisfactory results while reducing household costs and saving medical resources (Xie et al.).

Wang et al. analyzed the clinical characteristics and therapeutic effects of CAR-T cell therapy and Blinatumomab in the treatment of children with relapsed/refractory leukemia, and found that the duration of mitigation is limited. (Wang et al.).

With the expansion of the use of Blinatumomab, further research is needed to determine the mechanism of Blinatumomab to improve the long-term efficacy of immunotherapy. The current discovery of double-targeted antigen CAR-T cell therapy and CAR-T cell therapy combined with hematopoietic stem cell transplantation may lead to a breakthrough in CAR-T cells (10). For children with rapid progress or high-risk recurrence factors, Blinatumomab may be an ideal treatment for deeper relief and then bridging transplantation. In addition, CAR-T cell therapy may be more effective for children with extramedullary lesions. Considering many factors, CAR-T cell therapy and allogeneic CAR-T cell bridging HSCT after using Blinatumomab may be a new option. This topic provides some valuable clinical retrospective studies for the treatment of childhood leukemia. We invite you to read each of these enlightening articles.
